# Hepatic myelopathy neurological complication of chronic liver disease: two case reports

**DOI:** 10.1186/s13256-024-04495-2

**Published:** 2024-06-17

**Authors:** Mariem Mhiri, Mehdi Ben Abdelwahed, Mohamed Amine Dhiflaoui, Rihab Ben Dhia, Narjes Gouta, Imen Jemni, Raoua Baklouti, Mejda Zakhama, Arwa Gueddiche, Mohamed Hichem Loghmari, Nabil Ben Chaabene, Leila Safer, Mahbouba Frih-Ayed

**Affiliations:** 1 Departement of Neurology, CHU Fattouma Bourguiba, Fattouma Bourguiba Hospital, Avenue 1Er Juin. 5000, Monastir, Tunisia; 2 Departement of Gastro-Enterology, CHU Fattouma Bourguiba, Monastir, Tunisia

**Keywords:** Hepatic myelopathy, Shunt, Spastic paraparesis

## Abstract

**Background:**

Hepatic myelopathy is a very rare neurological complication of chronic liver disease. Patients habitually present with progressive pure motor spastic paraparesis. This neurological dysfunction is almost always due to cirrhosis and portocaval shunt, either surgical or spontaneous.

**Cases report:**

We report two cases of a 57-year-old man and a 37-year-old woman with progressive spastic paraparesis linked to cirrhosis and portal hypertension. The two patients are of Tunisian origin (north Africa). Magnetic resonance imaging of the spinal cord of two patients was normal, while brain magnetic resonance imaging showed a T2 hypersignals of the pallidums. These signs, in favor of hepatic encephalopathy in the two patients with cirrhosis with isolated progressive spastic paraparesis without bladder or sensory disorders, help to retain the diagnosis of hepatic myelopathy.

**Conclusion:**

Hepatic myelopathy is a severe and debilitating neurological complication of chronic liver disease. The pathogenesis is misunderstood and seems to be multifactorial, including the selective neurotoxic role both of ammonia and other pathogenic neurotoxins. Usually a pathological brain magnetic resonance imaging showing a hepatic encephalopathy was documented, contrasting with a normal spinal cord magnetic resonance imaging that contributed to diagnosis of hepatic myelopathy. Conservative therapies such as ammonia-lowering measures, diet supplementation, antispastic drugs, and endovascular shunt occlusion show little benefit in improving disease symptoms. Liver transplantation performed at early stage can prevent disease progression and could probably allow for recovery.

## Introduction

Neurological complications are common in patients with cirrhosis, affecting both central and peripheral nervous systems. Neurologic symptoms are variable, ranging from mild symptoms to sever encephalopathy with coma. Hepatic myelopathy is a very rare neurological complication of chronic liver disease causing progressive gait disorder, spastic paraparesis, and pyramidal syndrome [1]. Severe liver failure may be associated to cirrhosis and portocaval shunt, either surgical or spontaneous. The neurological symptoms are usually difficult to detect in clinical practice, especially during the onset period. In medical literature, only a few cases of hepatic myelopathy have been reported [2, 3].

In this work, we report two cases of progressive spastic paraparesis related to cirrhosis with portal hypertension. The physiopathology of the disease and the imaging features are discussed.

## Patient and observation

### Case 1

Patient information: a 57-year-old Tunisian patient (north Africa) presented to the neurology department with history of difficulty in walking with weakness and stiffness in both lower limbs. The weakness gradually progressed over the past 6 months. There was no history of sensory impairment and symptoms of bowel and bladder involvement were absent.

His medical history revealed liver cirrhosis related to Budd–Chiari syndrome with partial thrombosis of the inferior vena cava and superior right hepatic vein. Etiological investigation was negative and the patient was put on anti-vitamin K.

Clinical finding: at admission he did not complain of headaches, fever, or fasciculations. Systematic examination was unremarkable and neurological examination found a spastic paraparesis with scissor walk. In addition, there is quadripyramidal syndrome, especially in lower limbs with brisk deep tendon reflexes, and bilateral Babinski sign. There was no disturbance of superficial or deep sensitivity and cranial nerves examination was normal.

Diagnostic assessment: laboratory investigations revealed bicytopenia, hypocholesterolemia, liver transaminases were normal (Alanin aminotransferase (ALT), Aspartate aminotransferase (AST) (15/12 IU/l), total bilirubin/direct (10/5). Cerebrospinal fluid (CSF) examination without abnormalities (Table 1). Patient’s serum was no reactive to hepatitis A, B, C, lyme, and human immunodeficiency virus (HIV). Tests for a autoimmune diseases were negative, especially for antinuclear (AAN), Anti Neutrophil Cytoplasmic Antigen (ANCA), and aquaporin 4 antibodies.

**Table 1:** Hematological and biochemical profile of patients

	P1	P2
HB (G/Dl)	11.2	12
GR (10^6^)	4.11	5
WC(10^3^)	4.9	5.6
Platelets (10^3^)	125	91
Urea (mmol/l)	5	3
Creat (mmol/l)	63	57
ASAT	15	61
ALAT	12	46
Bili T (IU/l)	10	50
Bili direct (IU/l)	5	21
PAL	45	50
Cholesterolemia	2.8	2
TG	0.56	0.7
LDL-C	1.6	1.3
Serology HIV, HVB, HVC	0	HVB (positive, chronic)
Albumin (g/l)	27	21.8

HB: Hemoglobin, WC: white blood cells, creat: creatinin, ALT/AST: Alanin aminotransferase (ALAT), Aspartate aminotransferase (ASAT), PAl: alkaline phosphatase, TG: Triglycerides, LDL-c: LOW density of cholesterol, HVB: hepatitis virus B, HVC: Hepatitis virus C, HIV: Human immunosuppression virus

**Fig. 1:**
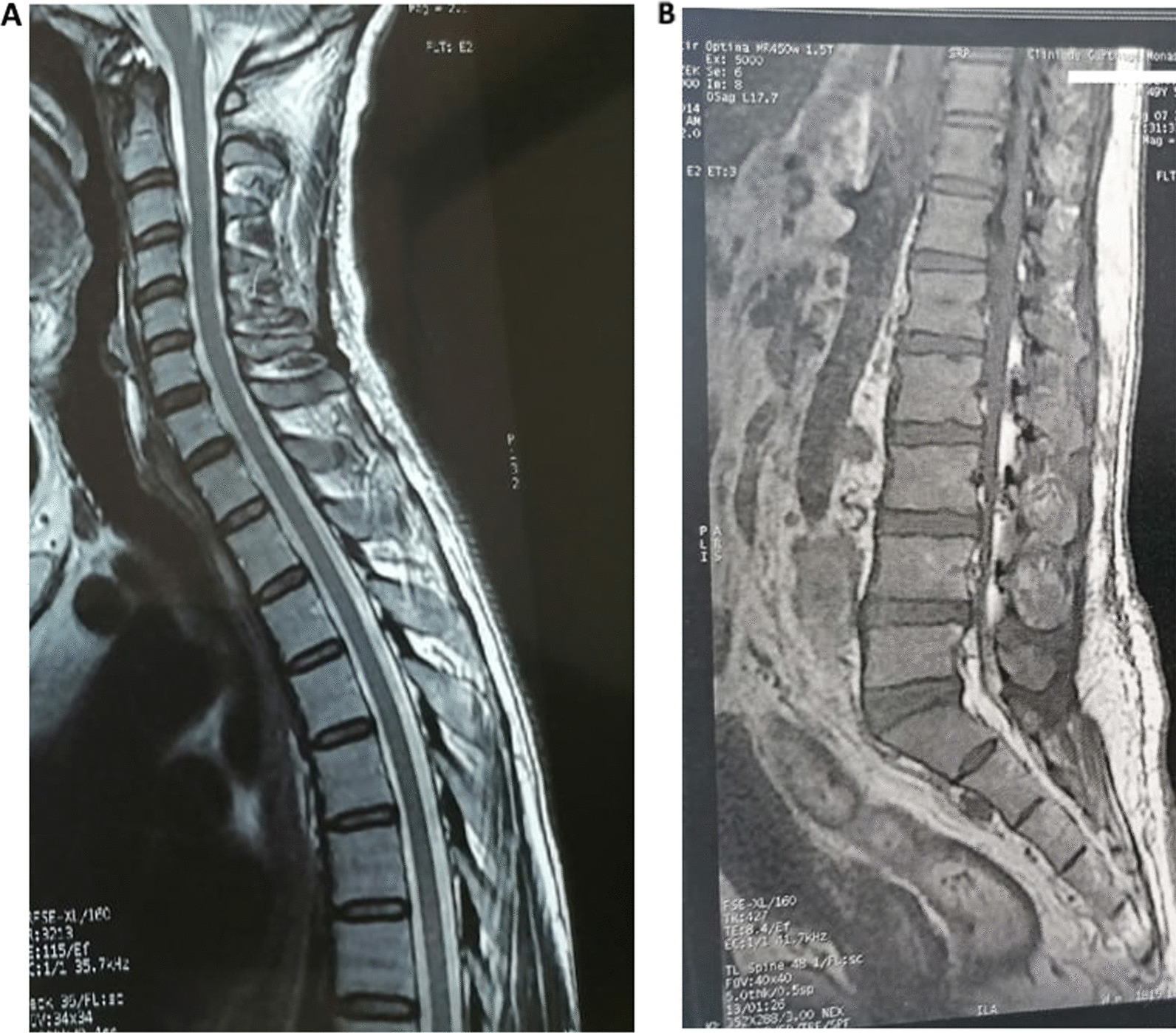
: Spinal cord magnetic resonance imaging (**A** T1 with Gado, **B** T2): No abnormalities

Medullar magnetic resonance imaging (MRI) noted the presence of multiple disco-vertebral degenerative phenomena staged on a narrow bipolar spinal canal without spinal cord compression or root conflict (Fig. 1).

**Fig. 2:**
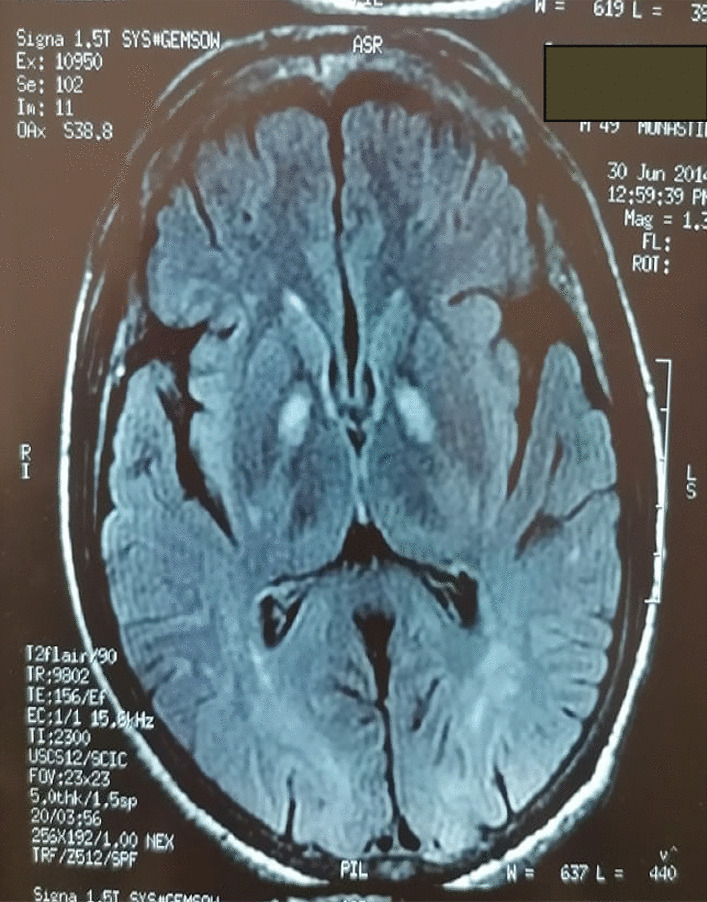
: Cerebral magnetic resonance imaging: axial slice, seq T2 Flair: bi-pallidal hypersignal

Cerebral MRI T2 showed a bilateral and symmetric hypersignal bipallidal (Fig. 2).

Diagnosis: hepatic myelopathy caused by a spontaneous portocaval shunt in the context of Budd–Chiari syndrome.

Therapeutic interventions: the patient received conservative therapies with antispastic drugs and motor reeducation.

Patient perspectives: a stabilization of the handicap.

Informed consent: applicable.

### Case 2

Patient information: a 37-year-old Tunisian patient (north Africa) presented to the neurology department for progressive walking disorders and sensation of heavy legs without sensitivity disorders. Her medical past history included Hodgkin’s lymphoma treated with radiochemotherapy in 2013 and viral B cirrhosis since 2017.

Clinical finding: neurological examination showed spastic paraplegia. Quadripyramidal syndrome especially in lower limbs with lively diffuse tendon reflexes, a bilateral Babinski sign, and clonus of the lower limbs. No sensitivity disorders or vesicosphincter disorders were noted.

Diagnostic assessment: her biological assessment showed hepatic failure with thrombocytopenia: 91,300, low TP: 35.5%; total bilirubin: 50 IU/l; low albumin level at 21.8 g/dl;  Alanin aminotransferase (ALT), Aspartate aminotransferase (AST): 61/46 IU/l and hyperammoniemia (81.11 mmol/l) (Table 1).

Medullary MRI was normal and cerebral MRI showed T2 hypersignals of the pallidums.

Diagnosis: as infectious, autoimmune, paraneoplasic, and compressive cause of myelopathies were eliminated, the diagnosis of hepatic myelopathy was made

Therapeutic interventions: the patient was treated with baclofen, vitamins, and nutritional supplementation, as well as motor rehabilitation and listed for liver transplantation.

Patient perspectives: on follow-up after 3 months, we noted a partial improvement.

Patient consent: applicable.

## Discussion

A few cases of hepatic myelopathy have been reported in medical literature and constitute an underestimated etiology of spastic paraparesis. The onset of hepatic myelopathy is insidious and usually occurs after surgical or spontaneous portosystemic shunts. The disease is characterized clinically by gait disorder, spastic paraparesis, and pyramidal syndrome without bladder or sensitive disorders [4].

Thoracic spinal cord involvement is more common and spinal cervical cord involvement is rarely seen [5]. Hepatic myelopathy with quadriparesis is rarely reported in the literature (KORI).

Histological studies performed in patients who died with hepatic myelopathy show symmetric demyelinization along the pyramidal tract, especially affecting the dorsal spinal cord. Axonal damage occurs habitually with the progression of the disease. In some cases, demyelinization is noted in the ventral pyramidal tracts in the posterior columns and spinocerebellar tracts [6, 7]. The physiopathology of these cord lesions occurring in patients with cirrhosis is still poorly understood and seems to be multifactorial disease. Toxic, hemodynamic, metabolic, and nutritional factors are incriminated [6]. The toxic impact of the ammonia and other neurotoxins on  the spinal cord is well known and can be explained by hepatic insufficiency and important spontaneous or surgical portocaval shunt [5, 6]. Impairment of liver function is also responsible of deficiencies of essential nutrients for the nervous system. Furthermore, hepatic myelopathy was reported in patients without liver cirrhosis [8].

The diagnostic of hepatic myelopathy is habitually difficult, especially at the early onset of symptoms, and diagnosis is usually made after exclusion of other causes of spastic paraparesia. Differential diagnosis of hepatic myelopathy includes amyotrophic lateral sclerosis, multiple sclerosis, and vascular and infectious spinal cord diseases. As in our patients, spinal cord MRI is normal in most cases. Indeed, the normality of spinal cord MRI constitutes a strong argument supporting the diagnosis of hepatic myelopathy. Cerebral MRI images showing signs of hepatic encephalopathy in the two patients with cirrhosis with isolated progressive spastic paraparesis without bladder or sensory disorders helped to make the diagnosis of hepatic myelopathy. Cerebral MRI may be normal or show signal abnormalities such as this T2 hypersignal type of involvement of the basal ganglia that was the same in our case [3, 9].

Neurophysiology tests constitute a valuable tool in early diagnostic of hepatic myelopathy even before symptom onset. The motor evoked potentials (MEP) induced by transcranial magnetic stimulation show, in most cases, a prolonged central motor conduction time [6].

Hepatic myelopathy is progressive and difficult to cure unless treatment has been instituted early. Conservative therapies including ammonia-lowering measures, diet supplementation, antispastic drugs, and endovascular shunt occlusion show little benefit in improving disease symptoms. Liver transplant could fully reverse the effects of hepatic myelopathy in patients with early disease, whereas it has little effect in patients with advanced disease [9, 10]. Interventional endovascular shunt occlusion can also be proposed [6, 11].

Axonal loss seems to be determining factor [12], and fecal transplantation seems to be an interesting therapeutic option [13].

## Conclusion

Hepatic myelopathy is a rare neurological complication of chronic liver disease. The diagnosis of the disease is based on clinical symptoms and the exclusion of other causes of spastic paraparesia. The diagnosis of hepatic myelopathy must be made as soon as possible before the occurrence of irreversible spine cord lesions. Motor evoked potentials play a crucial role in the diagnosis of the disease even before the onset of clinical signs. Conservative therapies have a limited effect and liver transplantation performed at early stage can prevent disease progression.

## Data Availability

Authors confirm that data are available when necessary.
